# Functional nanomaterials for immunomodulation in bone regeneration: a bibliometric analysis of the osteoimmune research landscape

**DOI:** 10.3389/fimmu.2026.1978326

**Published:** 2026-10-28

**Authors:** Yan-chen Feng, Zhe Zhang, Zi-yi Guo, Zhuo-yu Ren, Qiang Fu, Yin Fu, Ji Li

**Affiliations:** 1 School of Basic Medical Sciences, Heilongjiang University of Chinese Medicine, Harbin, Heilongjiang, China; 2 Department of First Clinical Medical College, Heilongjiang University of Chinese Medicine, Harbin, Heilongjiang, China; 3 National Engineering Laboratory for Internet Medical Systems and Applications, The First Affiliated Hospital of Zhengzhou University, Zhengzhou, Henan, China; 4 Department of Rehabilitation Medicine, The First Affiliated Hospital of Zhengzhou University, Zhengzhou, Henan, China; 5 Department of Anesthesiology, Pain and Perioperative Medicine, The First Affiliated Hospital of Zhengzhou University, Zhengzhou, Henan, China

**Keywords:** bibliometric analysis, bone regeneration, immunomodulation, macrophage polarization, nanomaterials, osseointegration, osteoimmunology

## Abstract

**Background:**

Functional nanomaterials are increasingly used in bone regeneration as both structural supports and bioactive interfaces that modulate the osteoimmune microenvironment. Macrophage regulation is a major research focus linking material properties with inflammation resolution, osteogenesis, angiogenesis, and osseointegration. This study aimed to characterize the scientific and intellectual landscape of nanomaterial-enabled bone regeneration from an osteoimmune perspective, with particular attention to macrophage-related research within the broader osteoimmune network.

**Methods:**

Publications addressing immune or osteoimmune regulation, nanomaterials, and bone regeneration or osseointegration were retrieved from the Web of Science Core Collection and Scopus through 31 December 2025. After deduplication and eligibility screening, 1,006 English-language original articles and reviews were included. Bibliometric analyses evaluated publication trends, collaboration, citation performance, keyword co-occurrence, conceptual structure, thematic evolution, reference co-citation, and citation-burst dynamics.

**Results:**

Annual output increased from 2 publications in 2012 to 291 in 2025, with 77.2% of records published during 2022–2025. Bone regeneration, osteogenesis, macrophage, immunomodulation, and macrophage polarization were the most prominent keywords. Network and thematic analyses showed close associations between immune regulation and angiogenesis, tissue engineering, hydrogels, nanoparticles, osseointegration, antibacterial strategies, and periodontitis. Co-citation and citation-burst analyses further indicated an evolving intellectual foundation linking macrophage biology, material-interface properties, and skeletal regeneration.

**Conclusion:**

The field is increasingly shifting from predominantly osteogenic material design toward nanomaterial-enabled regulation of the osteoimmune microenvironment. Macrophage-related research remains prominent, while spatiotemporal immune regulation, multicellular interactions, and disease-specific regenerative microenvironments are emerging priorities.

## 1 Introduction

Critical-sized and anatomically complex bone defects resulting from high-energy trauma, infection, tumor resection, and reconstructive surgery remain challenging because endogenous repair is often insufficient to restore skeletal architecture and mechanical function, particularly when vascular supply, soft-tissue integrity, and mechanical stability are simultaneously compromised (1). Advances in regenerative medicine have promoted the development of engineered constructs capable of recruiting endogenous cells, supporting tissue infiltration and vascularization, and providing spatially organized osteogenic cues (2, 3). Functional nanomaterials extend these strategies by introducing nanoscale surface features, hierarchical architectures, high interfacial area, and controlled delivery of bioactive ions or molecules, thereby enabling more precise regulation of cell–material interactions and the local regenerative microenvironment. Multiscale engineering of biodegradable Zn-based porous scaffolds has shown that material composition, nanoscale surface morphology, pore architecture, and degradation behavior can jointly regulate macrophage responses and osteogenesis (4). Hierarchical membranes incorporating nanofibrous or nanonetwork structures can similarly integrate space maintenance, antimicrobial activity, cellular infiltration, and osteogenic support within a single regenerative platform (5). Together, these studies illustrate how nanoscale material design can couple structural support with active biological regulation during bone regeneration.

Immune regulation is an integral component of the biological response to bone injury and material implantation. Among immune-cell populations, macrophages have received particular attention because their states and functions change dynamically across different phases of skeletal repair. Single-nucleus and multimodal analyses of fracture healing have identified temporally distinct macrophage populations associated with inflammatory, reparative, and inflammation-resolving phases, accompanied by coordinated changes in skeletal stem and progenitor cells (6). Temporal regulation is also evident during material-mediated regeneration: magnesium-associated bone repair involves an early macrophage-dependent TRPM7-mediated immunoregulatory response that promotes osteogenesis, whereas prolonged exposure can alter inflammatory and mineralization responses (7). Importantly, macrophage heterogeneity extends beyond the canonical M1/M2 classification. CD301b-positive macrophages have been identified as a regenerative population that supports periodontal bone formation (8), while periosteal Tenascin-C-mediated recruitment of inflammatory macrophages can promote skeletal stem-cell activation and bone regeneration (9). Macrophage differentiation can further influence osteoclastogenesis and H-type vessel formation, linking myeloid-cell states with bone resorption, vascular organization, and skeletal homeostasis (10). High-resolution studies have also shown that stromal populations participate in the early organization of immune-cell recruitment and tissue responses (11). Together, these findings place macrophage regulation within a broader osteoimmune network involving the spatial and temporal coordination of immune, stromal, vascular, and skeletal-cell populations during bone regeneration.

Nanoengineered materials can regulate osteoimmune interactions through multiple physicochemical and biological mechanisms. Immunomodulatory implant surfaces incorporating Zn²^+^ and osteoinductive signals can influence macrophage recruitment and osteogenic activity through controlled surface chemistry (12). Nanoparticle-containing scaffolds capable of sustained local hydrogen delivery can remodel the inflammatory and senescent microenvironment of aged bone by altering macrophage states and secretory activity (13). In infected bone defects, electroactive biomimetic mineralized scaffolds can integrate antibacterial activity with regenerative signaling (14), while engineered nanostructures with artificial antioxidase activity can regulate redox homeostasis and improve the regenerative microenvironment of inflammatory maxillofacial defects (15). Temporal regulation provides an additional design dimension: inflammation-responsive implant coatings can sequentially coordinate immune activity, angiogenesis, and osteogenesis during osseointegration (16), whereas multifunctional Janus membranes can combine immunomodulatory, barrier, antibacterial, and osteogenic functions during guided bone regeneration (17). Collectively, these strategies demonstrate how nanomaterial-enabled systems can couple surface chemistry, therapeutic delivery, redox regulation, and temporal control with immune and regenerative responses during bone repair.

Previous bibliometric studies have broadly mapped bone-regeneration materials, including scaffolds, hydrogels, extracellular-matrix-related strategies, and drug-delivery systems (18). A more focused analysis examined macrophage polarization in nanomaterial-associated osteogenesis (19), whereas another mapped immunomodulatory biomaterials primarily in the context of implant osseointegration (20). These studies have characterized important but partly distinct segments of the literature, leaving the broader connections among nanomaterial engineering, osteoimmune regulation, macrophage-related research, osteogenesis, angiogenesis, osseointegration, and disease-specific regenerative settings less clearly resolved. The continuing growth and diversification of the literature also provide an opportunity to examine how these themes have become interconnected over time. Accordingly, the present study integrates records from the Web of Science Core Collection and Scopus to characterize the scientific and intellectual landscape of nanomaterial-enabled bone regeneration from an osteoimmune perspective. We analyze publication and collaboration patterns, keyword co-occurrence and conceptual structure, thematic evolution, reference co-citation, and citation-burst dynamics, with particular attention to macrophage-related research as a prominent cellular axis within the broader osteoimmune network. This integrated framework is intended to clarify the development, convergence, and emerging priorities of nanomaterial-enabled osteoimmunomodulation in bone regeneration.

## 2 Methods

### 2.1 Data sources and search strategy

A systematic literature search was conducted in the Web of Science Core Collection (WoSCC) and Scopus databases. The final database searches and data exports were completed on 6 May 2026, with the publication period restricted to records published through 31 December 2025.

The search strategy was structured around three complementary dimensions of the research scope: biological regulation, material platform, and regenerative application. Accordingly, three principal concept domains were defined: immune or osteoimmune regulation, nanomaterials, and bone regeneration or osseointegration. The immune/osteoimmune domain included broad immunoregulatory and osteoimmune terms together with selected macrophage-related expressions. The nanomaterial domain defined the material scope of the analysis, whereas the bone regeneration/osseointegration domain represented the skeletal regenerative application and encompassed both bone repair and implant–bone interface integration. Terms within each concept domain were combined using the Boolean operator “OR,” and the three domains were combined using “AND” to retrieve publications addressing all three dimensions. This structure maintained a specific focus on nanomaterial-enabled systems while retaining broad coverage of immune regulatory mechanisms and skeletal regenerative settings. Relevant synonyms, spelling variants, and related expressions were incorporated within each domain. The complete database-specific Boolean search strings, including field tags and initial retrieval counts, are provided in **Supplementary Material Sections S1.1, S1.2**.

In WoSCC, the search was performed using the Topic field (TS), which covers titles, abstracts, author keywords, and Keywords Plus. In Scopus, the corresponding search was conducted using the TITLE-ABS-KEY field. No restrictions on language or document type were applied at the initial retrieval stage. Full bibliographic records and cited references were exported from WoSCC in plain-text format, whereas bibliographic records were exported from Scopus in CSV format. The exported metadata included titles, abstracts, author names, institutional affiliations, countries or regions, source titles, publication years, document types, keywords, and citation information.

### 2.2 Literature screening and data processing

Records retrieved from WoSCC and Scopus were merged into a single bibliographic dataset. Duplicate records were initially identified by exact DOI matching. For records without a DOI, potential duplicates were identified by comparing article titles, author names, source titles, and publication years. Suspected duplicate records were manually reviewed before removal to confirm that they represented the same publication. The numbers of records retained and excluded at each stage of the selection process are provided in **Figure 1**; **Supplementary Material Section S2**.

**Figure 1:**
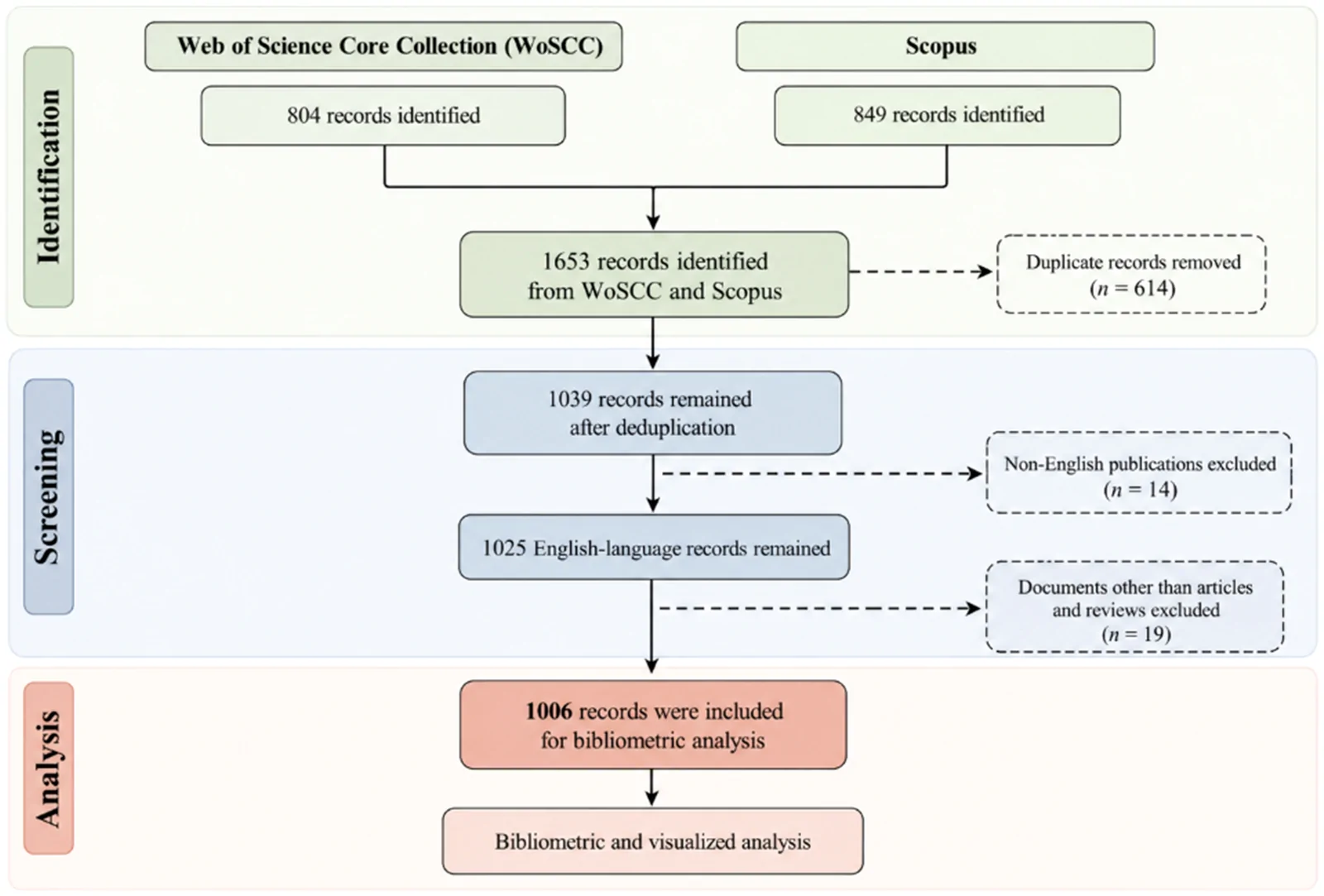
PRISMA-style flow diagram of literature identification, deduplication, screening, and final inclusion.

Two investigators independently assessed the deduplicated records for eligibility. Publications were eligible for inclusion if they were written in English and classified as original research articles or reviews. Records published in other languages or classified as other document types were excluded. Disagreements regarding eligibility were resolved through discussion, with consultation of a third investigator when consensus could not be reached. The overall study-selection process is summarized in a PRISMA-style flow diagram (**Figure 1**).

Before bibliometric analysis, author keywords were harmonized using a manually curated thesaurus. Synonymous terms and obvious lexical variants were consolidated under a single preferred term, whereas conceptually related but non-equivalent terms were retained separately. For example, “macrophage polarisation” was standardized as “macrophage polarization,” and “nano-hydroxyapatite” was standardized as “nanohydroxyapatite”.

### 2.3 Bibliometric and visualization analysis

Bibliometric and visualization analyses were conducted using the Bibliometrix package (v4.1) in R, VOSviewer (v1.6.19), CiteSpace (v6.2), and Python (v3.9). Annual publication trends and the productivity of countries, institutions, journals, and authors were evaluated using publication and citation indicators. National productivity was assigned according to the corresponding author’s country, whereas country- and institution-level collaboration patterns were derived from author-affiliation data. International collaboration was defined as publications involving affiliations from at least two countries. Author productivity was assessed using both full and fractional counting.

The 2025 Journal Impact Factors (JIFs) and corresponding JCR category quartiles were obtained from the 2026 edition of Clarivate Journal Citation Reports (JCR). Citation performance was evaluated using total citations, average citations per publication, and dataset-based h-indices calculated from the included records.

Keyword co-occurrence, conceptual structure, thematic mapping, and thematic evolution analyses were performed using the harmonized author keywords described in Section 2.2. Conceptual structure was examined using multiple correspondence analysis (MCA). Thematic evolution was evaluated across three periods (2012–2018, 2019–2021, and 2022–2025). Keyword co-occurrence networks were visualized using VOSviewer, with node size representing keyword occurrence frequency and link thickness reflecting co-occurrence strength.

Annual frequencies of selected high-frequency author keywords were analyzed using the Prediction function of the Temporal Analysis module in BiblioVista (version 1.0.0). Observed annual frequencies from 2012 to 2025 were used to generate projections through 2030, with forecast uncertainty represented by prediction intervals. The resulting projections were interpreted as directional estimates of temporal trends rather than precise predictions.

Reference co-citation analysis was based on cited references extracted from the WoSCC cited-reference (CR) field. Cited references were standardized using DOI information or, when unavailable, bibliographic identifiers constructed from the first author, publication year, source title, volume, and starting page. Reference communities were identified using the weighted Louvain community-detection algorithm. For visualization, the core co-citation network comprised 63 references and the 250 strongest co-citation links. Citation bursts were detected in CiteSpace using Kleinberg’s burst-detection algorithm, and the 25 references with the strongest citation bursts during 2012–2025 were retained for analysis.

### 2.4 Sensitivity analysis

To assess the robustness of the combined-database approach, WoSCC and Scopus records were analyzed separately using the same eligibility criteria and keyword-harmonization procedures as those applied in the primary analysis. Before frequency ranking, synonymous terms and obvious lexical variants, including singular and plural forms, were consolidated under the same preferred terms. The 20 most frequent author keywords were identified independently for each database. Concordance between the two databases was evaluated by calculating the proportion of shared top-20 keywords and the Jaccard similarity coefficient. For keywords appearing in the top 20 of both databases, rank-order agreement was further assessed using Spearman’s rank correlation coefficient.

## 3 Results

### 3.1 Literature search and screening outcomes

The literature search identified 1,653 records, including 804 from the Web of Science Core Collection (WoSCC) and 849 from Scopus (**Figure 1**). After removal of 614 duplicates, 1,039 unique records remained. Screening excluded 14 non-English publications and 19 records that were neither articles nor reviews, resulting in a final dataset of 1,006 records for bibliometric and visualization analyses.

### 3.2 Annual publication trends

Annual publication output increased from 2 publications in 2012 to 291 in 2025, with 1,006 publications included overall (**Figure 2**). For descriptive purposes, the publication trajectory was summarized across three periods. During 2012–2018, annual output remained relatively low, increasing from 2 to 24 publications, with 53 publications produced cumulatively during this period. During 2019–2021, annual output increased from 25 to 96 publications, bringing the cumulative number of publications to 229 by 2021. During 2022–2025, annual output increased further from 118 to 291 publications. This period contributed 777 publications, accounting for 77.2% of the total dataset. The 291 publications published in 2025 alone exceeded the cumulative output during 2012–2018 (n = 53).

**Figure 2:**
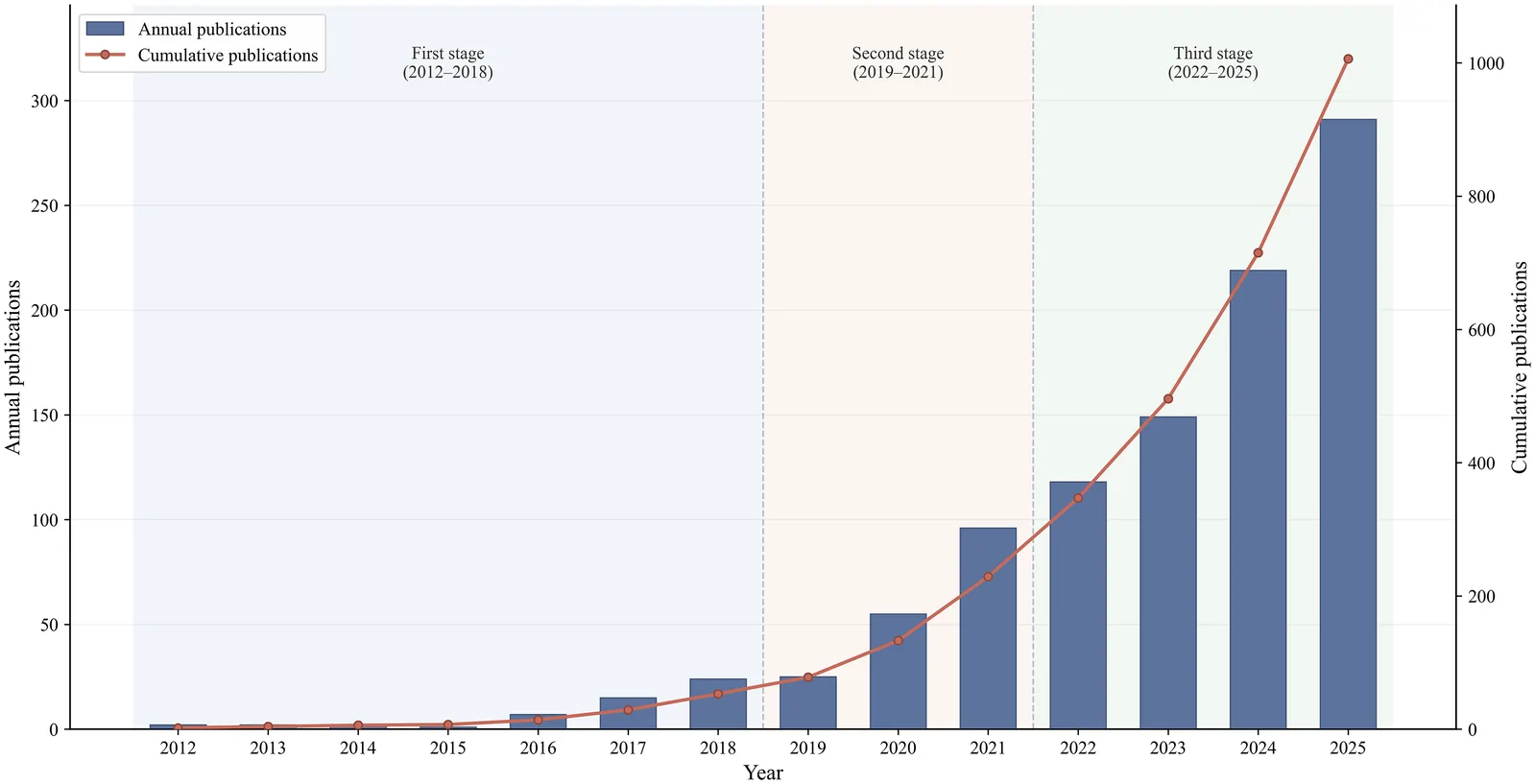
Annual publication trends from 2012 to 2025.

### 3.3 National productivity and international collaboration

**Table 1** summarizes national publication output according to the corresponding author’s country, whereas **Figure 3** depicts international collaboration based on all countries represented in the author affiliations. China ranked first in publication output, contributing 766 publications (76.1% of the 1,006 records) and receiving 31,421 citations. It also had the highest numbers of single-country publications (SCP = 662) and multiple-country publications (MCP = 104), with an MCP ratio of 0.136. The United States ranked second with 42 publications and had the highest average citation count among the 10 most productive countries (56.52 citations per publication), followed by Australia with 28 publications and an average of 53.00 citations per publication. Among the top 10 countries, the United Kingdom had the highest MCP ratio (0.625; 8 publications), followed by Australia (0.536), Spain (0.500), Iran (0.500), and the Netherlands (0.500), whereas India and Italy had MCP ratios of 0.143 and 0.200, respectively.

**Table 1:** The top 10 productive countries.

Country	Counts	Total citations	Avg. citations	SCP	MCP	MCP Ratio
China	766	31421	41.02	662	104	0.136
USA	42	2374	56.52	27	15	0.357
Australia	28	1484	53	13	15	0.536
India	21	537	25.57	18	3	0.143
South Korea	18	654	36.33	11	7	0.389
Spain	14	441	31.5	7	7	0.5
Iran	14	262	18.71	7	7	0.5
Italy	10	341	34.1	8	2	0.2
United Kingdom	8	321	40.12	3	5	0.625
Netherlands	8	136	17	4	4	0.5

SCP, Single Country Publications; MCP, Multiple Country Publications; Country, corresponding author’s country; Total Citations, The sum of global citations for the country’s publications included in this dataset; Avg. Citations, Average citations per paper (Total Citations/Articles).

**Figure 3:**
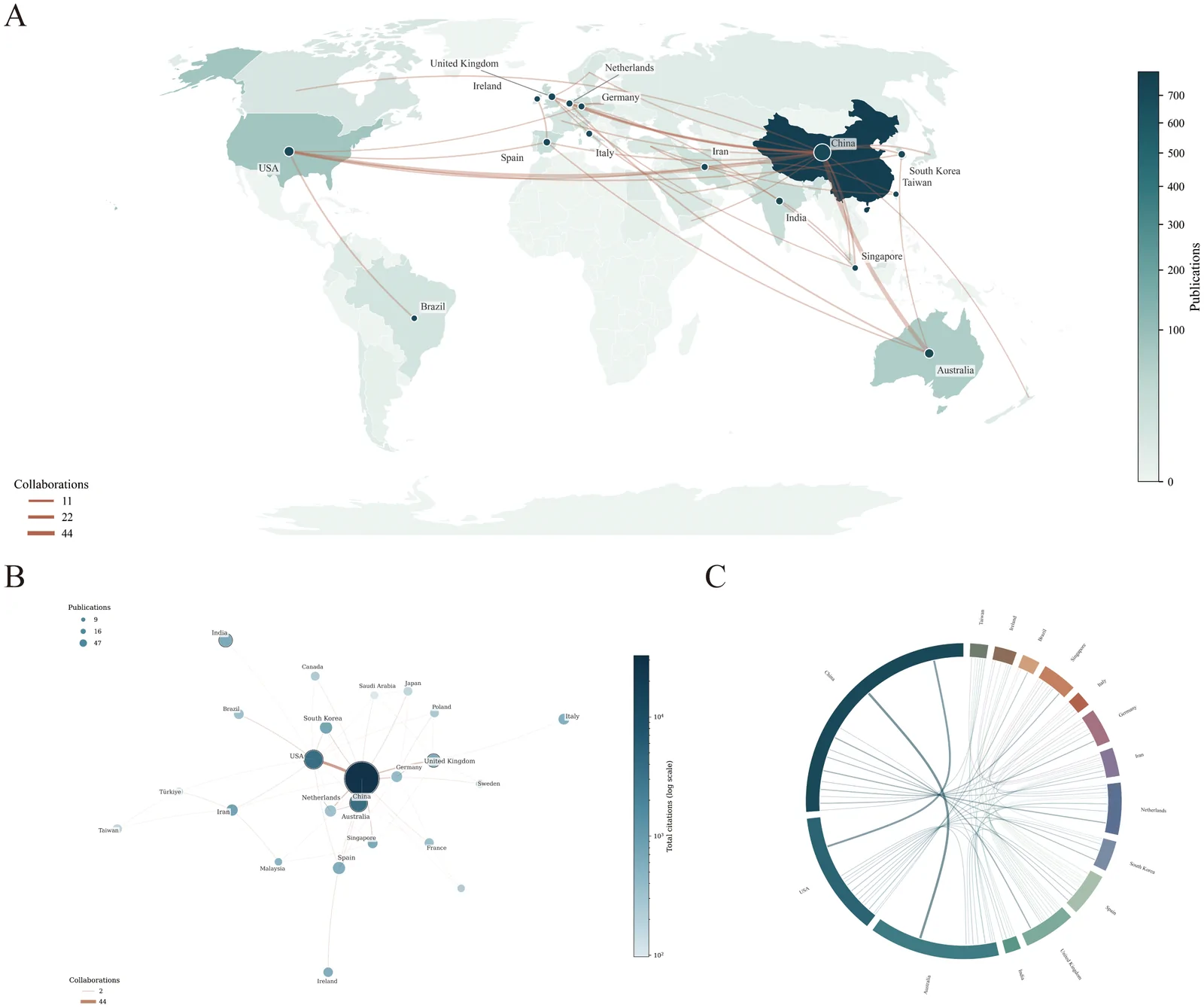
Global publication output and international collaboration patterns. **(A)** Geographical distribution of publications and international co-authorship links. Country shading and node size represent publication output, while line width indicates bilateral collaboration frequency. **(B)** Country collaboration network, in which node size represents publication count, node color indicates total citations on a logarithmic scale, and edge width represents collaboration frequency. **(C)** Chord diagram showing the collaboration structure among the 15 most productive countries; sector width represents total collaboration strength and chord thickness denotes bilateral collaboration frequency.

The geographical collaboration map showed that publication output was strongly concentrated in China, with collaborative links extending across North America, Europe, Asia, and Oceania (**Figure 3A**). The China–United States and China–Australia connections were among the most prominent bilateral links. In the collaboration network (**Figure 3B**), China formed the largest node, while the United States, Australia, the United Kingdom, Germany, South Korea, and Iran constituted additional visible nodes. The chord diagram (**Figure 3C**) showed that collaboration links were concentrated among a relatively limited group of countries.

### 3.4 Institutional productivity, citation performance, and collaboration

Shanghai Jiao Tong University was the most productive institution, with 104 publications, 5,185 total citations, an average of 49.86 citations per publication, and the highest dataset-based h-index among the top 10 institutions (h = 44) (**Table 2**). Sichuan University ranked second in publication output with 92 publications, followed by the Chinese Academy of Sciences with 90. The Chinese Academy of Sciences recorded higher total citations (4,653 vs. 3,980), average citations per publication (51.70 vs. 43.26), and h-index (39 vs. 36) than Sichuan University. Wuhan University had the highest average citation count among the top 10 institutions, with 76.73 citations per publication from 26 publications, followed by Huazhong University of Science and Technology with 64.47 citations per publication and an h-index of 22. Southern Medical University and Peking University contributed 32 and 26 publications, respectively, with average citation counts of 42.81 and 44.73 citations per publication.

**Table 2:** Top 10 most productive institutions and their citation indicators.

Institution	Counts	Total citations	Avg. citations	H-index
Shanghai Jiao Tong University	104	5,185	49.86	44
Sichuan University	92	3,980	43.26	36
Chinese Academy of Sciences	90	4,653	51.70	39
Air Force Medical University	36	1,707	47.42	20
Southern Medical University	32	1,370	42.81	18
Chongqing Medical University	31	1,551	50.03	18
Huazhong University of Science & Technology	30	1,934	64.47	22
Fudan University	29	1,275	43.97	16
Wuhan University	26	1,995	76.73	18
Peking University	26	1,163	44.73	17

The annual publication heatmap showed an increase in institutional publication output after 2020, with the highest annual outputs concentrated in 2024–2025 (**Figure 4A**). Shanghai Jiao Tong University, Sichuan University, and the Chinese Academy of Sciences published 22, 25, and 22 articles, respectively, in 2024, increasing to 28, 32, and 30 in 2025. Most other institutions maintained annual outputs below 10 publications during much of the study period, while Southern Medical University, Chongqing Medical University, and Fudan University showed increased publication activity from approximately 2017–2019 onward.

**Figure 4:**
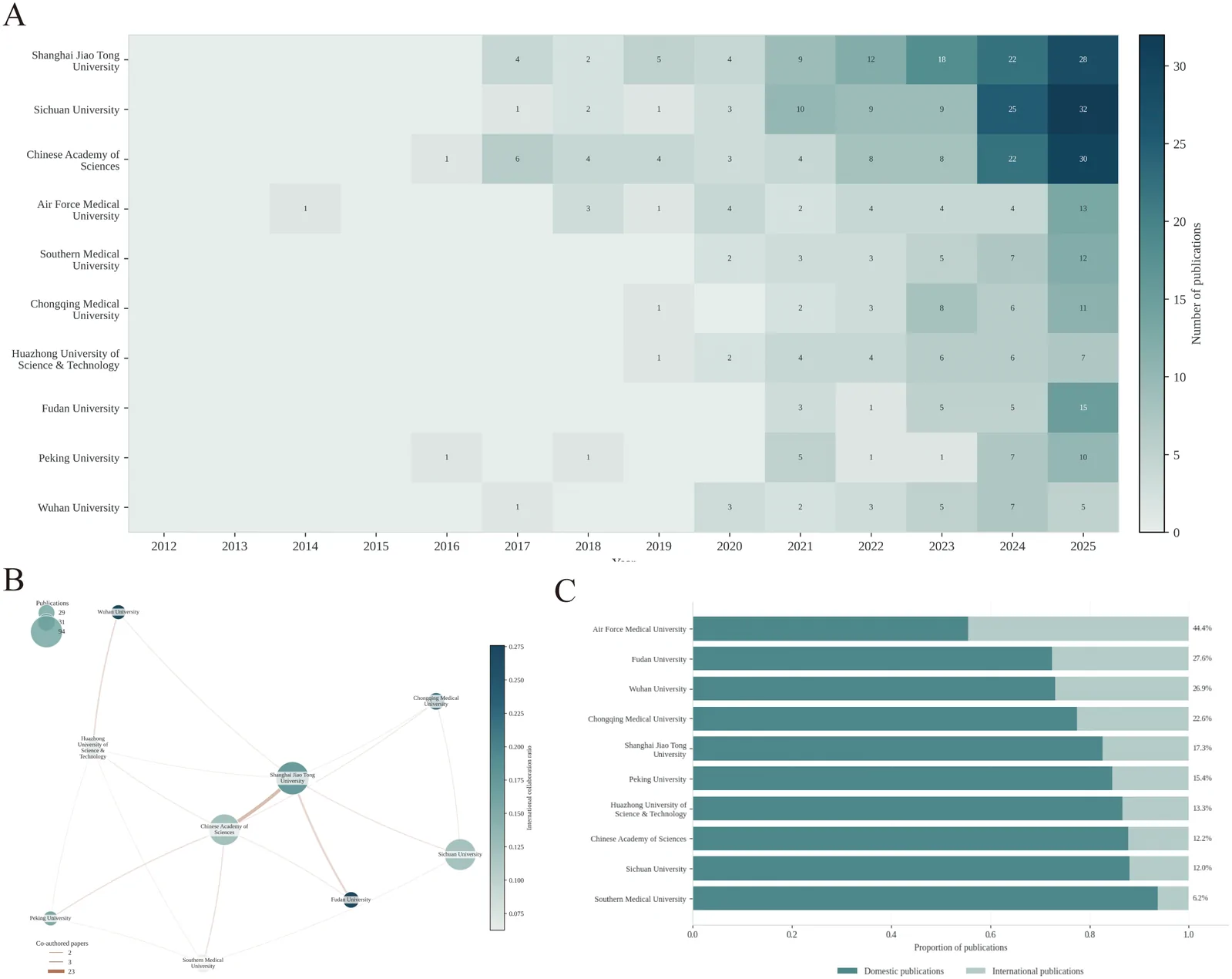
Temporal publication trends, collaboration structure, and internationalization of the leading institutions. **(A)** Annual publication output of the top 10 most productive institutions from 2012 to 2025. Cell color and the values within cells indicate the number of publications in each year. **(B)** Collaboration network among the leading institutions. Node size represents publication output, node color indicates the international collaboration ratio, and edge width denotes the number of co-authored publications between institutions. Only institutional collaboration links represented by at least two co-authored publications are shown. **(C)** Proportions of domestic and internationally co-authored publications among the top 10 institutions. International publications were defined as publications involving author affiliations from at least two countries, and the international collaboration ratio was calculated by dividing the number of international publications by the total number of publications.

In the institutional collaboration network, Shanghai Jiao Tong University formed the most prominent node and showed collaborative links with the Chinese Academy of Sciences, Fudan University, Sichuan University, Huazhong University of Science and Technology, and other institutions (**Figure 4B**). Its strongest visible co-authorship link was with the Chinese Academy of Sciences, which also formed a prominent node in the network.

International collaboration ratios varied across the leading institutions (**Figure 4C**). Air Force Medical University had the highest ratio, with 16 of 36 publications involving affiliations from at least two countries (44.4%). Fudan University, Wuhan University, and Chongqing Medical University had corresponding ratios of 27.6%, 26.9%, and 22.6%, respectively, whereas Southern Medical University had the lowest ratio at 6.2%. Shanghai Jiao Tong University, the Chinese Academy of Sciences, and Sichuan University had international collaboration ratios of 17.3%, 12.2%, and 12.0%, respectively.

### 3.5 Journal productivity, citation performance, and temporal trends

The 10 most productive journals published 377 articles, accounting for 37.5% of the 1,006 records included in the analysis (**Table 3** and **Figure 5A**). Bioactive Materials ranked first with 59 articles, 4,443 total citations, and an average of 75.31 citations per article. It also had the highest 2025 Journal Impact Factor among the journals listed in **Table 3** (IF = 23.6; JCR Q1). Advanced Healthcare Materials and Materials Today Bio ranked second and third in publication output, with 51 and 45 articles, respectively, followed by the Journal of Nanobiotechnology (39 articles) and the Chemical Engineering Journal (37 articles). All 10 of the most productive journals were classified as JCR Q1.

**Table 3:** Top 10 most productive journals and their citation indicators.

Journal	Counts	Total citations	Avg. citations	IF2025	JCR2025
Bioactive Materials	59	4443	75.31	23.6	Q1
Advanced Healthcare Materials	51	1714	33.61	11.0	Q1
Materials Today Bio	45	938	20.84	11.0	Q1
Journal Of Nanobiotechnology	39	1572	40.31	15.0	Q1
Chemical Engineering Journal	37	882	23.84	12.5	Q1
Biomaterials	32	4057	126.78	13.6	Q1
Acs Applied Materials & Interfaces	31	1259	40.61	7.8	Q1
Acs Nano	29	1848	63.72	17.3	Q1
International Journal Of Nanomedicine	29	1359	46.86	8.7	Q1
International Journal Of Biological Macromolecules	25	474	18.96	8.7	Q1

**Figure 5:**
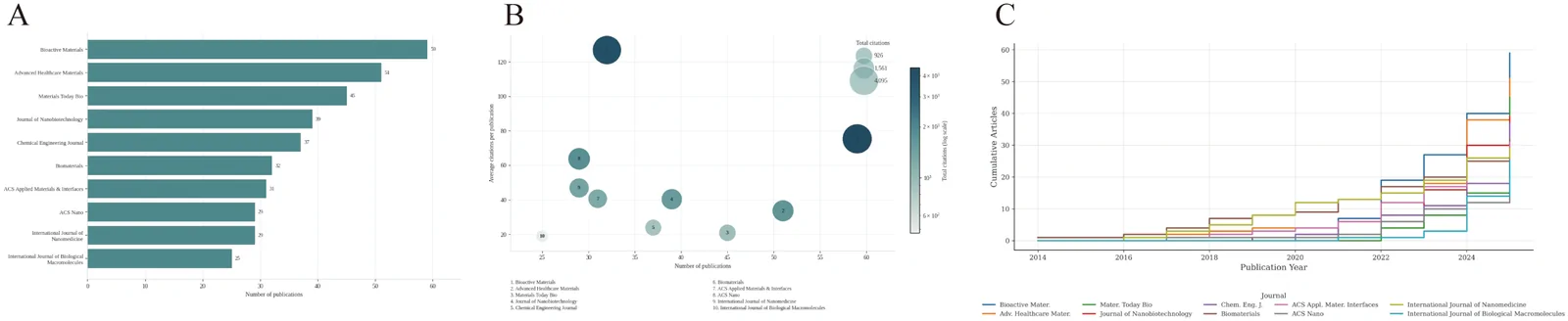
Productivity, citation performance, and cumulative publication trajectories of the leading journals. **(A)** Publication output of the 10 most productive journals. Bar length represents the number of publications, with exact publication counts shown at the end of each bar. **(B)** Relationship between journal productivity and citation performance. The horizontal axis represents the number of publications, the vertical axis represents the average number of citations per publication, and bubble size and color indicate total citations on a logarithmic scale. Numbers within the bubbles correspond to the journal list shown below the plot. **(C)** Cumulative publication trajectories of the top 10 productive journals. Each step represents publications added during the corresponding year, and steeper segments indicate periods of more rapid growth in publication output.

Citation metrics varied across journals (**Figure 5B**). Biomaterials, which ranked sixth in publication output with 32 articles, had the highest average citation count among the top 10 journals (126.78 citations per article) and accumulated 4,057 total citations. Bioactive Materials had the highest publication output and total citation count and the second-highest average citation count. ACS Nano received 1,848 citations from 29 articles, corresponding to an average of 63.72 citations per article. Materials Today Bio and the Chemical Engineering Journal published 45 and 37 articles, respectively, with average citation counts of 20.84 and 23.84 citations per article.

The cumulative publication trajectories showed differences in the timing and rate of journal contributions (**Figure 5C**). Biomaterials was among the earliest journals represented in the dataset and accumulated publications throughout the study period. The International Journal of Nanomedicine and Advanced Healthcare Materials also contributed from the mid-2010s onward. Bioactive Materials showed a marked increase after 2020 and reached the highest cumulative publication count by 2025, with 19 articles published in 2025. Advanced Healthcare Materials published 20 articles in 2024 and 13 in 2025. Materials Today Bio recorded 30 articles in 2025, while the Chemical Engineering Journal and ACS Nano published 19 and 17 articles, respectively, in the same year. Citation metrics reported here were calculated within the analyzed dataset and were not normalized by publication year or citation window.

### 3.6 Author productivity, citation performance, collaboration, and temporal trends

Xiao, Yin was the most productive author, with 28 publications, 2,626 total citations, and an average of 93.79 citations per publication (**Table 4**). Xiao, Yin also had the highest dataset-based h-index (h = 21), fractionalized publication count (3.87), and total link strength (219) among the 10 most productive authors. Ivanovski, Saso ranked second with 11 publications, followed by Liu, Xuanyong and Xiao, Lan with 10 publications each. The remaining six authors contributed 8–9 publications each.

**Table 4:** Top 10 most productive authors.

Author	Publications	Fractionalized publications	Total citations	Average citations	h-index	Total link strength
Xiao, Yin	28	3.87	2,626	93.79	21	219
Ivanovski, Saso	11	2.62	483	43.91	9	44
Liu, Xuanyong	10	1.09	423	42.30	8	94
Xiao, Lan	10	1.48	409	40.90	9	74
Bai, Long	9	0.94	885	98.33	9	88
Zhou, Zongke	9	0.76	345	38.33	8	107
Li, Jidong	9	1.04	305	33.89	7	72
Jin, Shue	9	0.96	297	33.00	8	79
Zheng, Kai	9	1.16	270	30.00	8	67
Cai, Lin	8	0.88	1126	140.75	8	69

Citation metrics varied considerably among authors with similar publication outputs. Cai, Lin, who contributed 8 publications, had the highest average citation count among the top 10 authors, at 140.75 citations per publication, with 1,126 total citations. Bai, Long received 885 citations from 9 publications, corresponding to an average of 98.33 citations per publication. The average citation counts of the other leading authors ranged from 30.00 to 93.79 citations per publication. In addition to Xiao, Yin (total link strength = 219), relatively high total link strengths were observed for Zhou, Zongke (107), Liu, Xuanyong (94), Bai, Long (88), and Jin, Shue (79).

The temporal distribution of publications showed heterogeneous activity patterns among the leading authors (**Figure 6**). Xiao, Yin published across most years from 2016 to 2025, with a peak annual output of six publications in 2021. Ivanovski, Saso and Liu, Xuanyong also showed publication activity across multiple years, with Liu, Xuanyong publishing four articles in 2025. Xiao, Lan and Bai, Long were active from approximately 2018–2020 onward. By contrast, most publications by Zhou, Zongke, Li, Jidong, Jin, Shue, Zheng, Kai, and Cai, Lin occurred after 2021. Zhou, Zongke published five articles in 2024, while Li, Jidong and Zheng, Kai each recorded four publications in 2025 and 2024, respectively.

**Figure 6:**
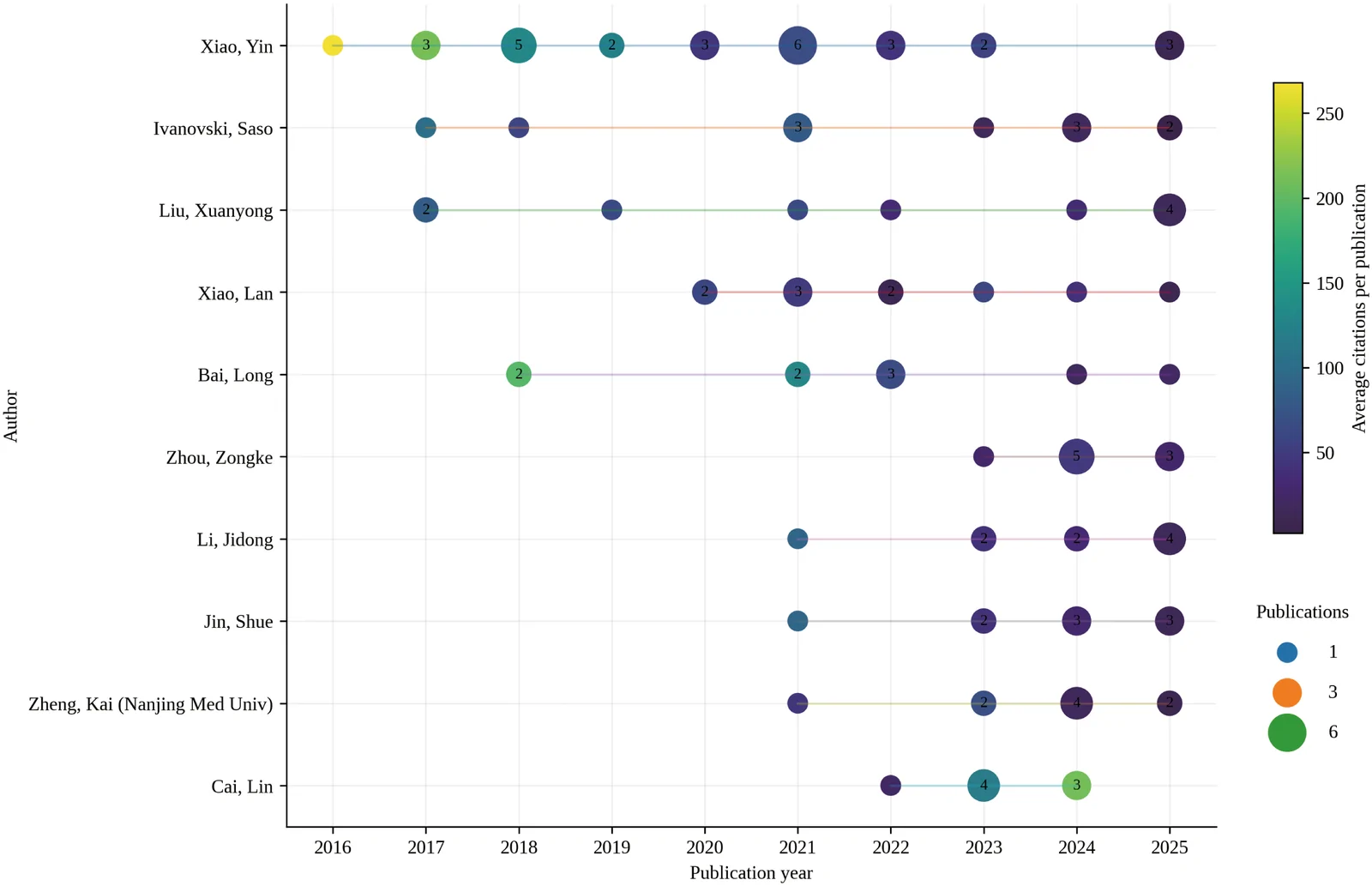
Annual publication activity of the top 10 productive authors.

### 3.7 Keyword distribution, co-occurrence structure, temporal trends, and projected trajectories

The treemap showed that bone regeneration was the most frequently occurring keyword, with 171 occurrences, accounting for approximately 10% of all keyword occurrences (**Figure 7A**). This was followed by osteogenesis (127; 7%), macrophage (119; 7%), immunomodulation (117; 7%), and macrophage polarization (112; 6%). Other frequently occurring keywords included osseointegration (77; 4%), osteoimmunomodulation (66; 4%), hydrogel (49; 3%), and periodontitis (47; 3%). Additional recurrent keywords included tissue engineering, angiogenesis, antibacterial, biomaterial, inflammation, osteogenic differentiation, nanoparticle, exosomes, and extracellular vesicles.

**Figure 7:**
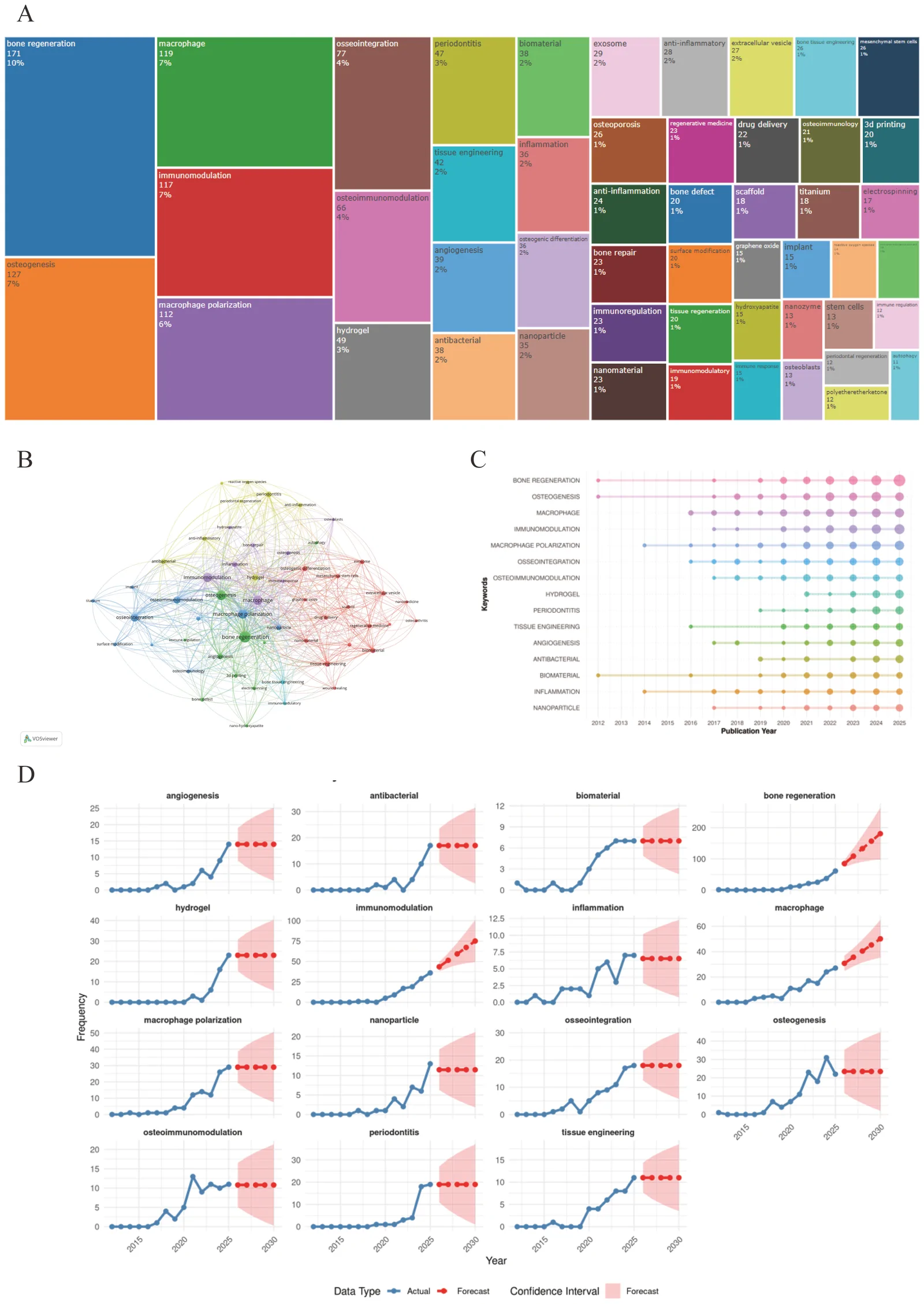
Keyword distribution, co-occurrence structure, temporal dynamics, and projected research trends. **(A)** Treemap of the most frequently occurring keywords. Rectangle size represents keyword frequency, and the percentage indicates the proportion of total keyword occurrences. **(B)** Keyword co-occurrence network. Each node represents a keyword, node size is proportional to its occurrence frequency, link thickness indicates co-occurrence strength, and node colors denote thematic clusters. **(C)** Temporal distribution of the leading keywords from 2012 to 2025. Bubble position indicates the year of occurrence, bubble size reflects annual keyword frequency, and connecting lines illustrate the continuity of each topic over time. **(D)** Observed and forecasted annual frequencies of selected high-frequency keywords. Blue lines and points represent observed frequencies, red lines and points indicate projected values, and the shaded areas show the corresponding prediction intervals. Forecasts should be interpreted as directional estimates because uncertainty increases with the prediction horizon.

The keyword co-occurrence network showed that bone regeneration, osteogenesis, macrophage, immunomodulation, and macrophage polarization formed large, highly connected nodes (**Figure 7B**). These keywords showed multiple co-occurrence links with angiogenesis, antibacterial, hydrogel, tissue engineering, nanoparticle, and osseointegration. The network further comprised several interconnected keyword clusters, with links observed among terms related to implant integration, inflammation, tissue regeneration, nanoscale delivery systems, and periodontal applications.

The temporal distribution showed differences in the timing and frequency of keyword occurrence (**Figure 7C**). Biomaterial and inflammation appeared relatively early in the study period, whereas macrophage polarization, macrophage, osseointegration, tissue engineering, angiogenesis, and nanoparticle became more frequent from the mid-to-late 2010s onward. Osteoimmunomodulation, hydrogel, and periodontitis occurred more prominently during the later years of the study period. Larger annual node sizes were observed for several major keywords during 2022–2025, including bone regeneration, osteogenesis, immunomodulation, macrophage polarization, and osseointegration.

Exploratory time-series projections showed different temporal trajectories across the selected keywords (**Figure 7D**). Within these projections, bone regeneration showed the largest increase and remained the most frequent keyword through 2030, while increasing trajectories were also observed for immunomodulation and macrophage. By contrast, angiogenesis, antibacterial, biomaterial, hydrogel, inflammation, macrophage polarization, nanoparticle, osseointegration, osteogenesis, osteoimmunomodulation, periodontitis, and tissue engineering were projected to remain closer to their recent frequency levels. Prediction intervals widened with increasing forecast horizon, particularly toward 2030, indicating greater uncertainty in the longer-term projections.

Database-specific sensitivity analyses showed substantial concordance in the leading author-keyword patterns between WoSCC and Scopus (**Supplementary Material S4**). Nineteen of the 20 most frequent author keywords were shared between the two databases, corresponding to a top-20 overlap of 95% and a Jaccard similarity coefficient of 0.905. The rankings of the shared keywords were positively correlated (Spearman’s ρ = 0.802, P < 0.001), indicating substantial consistency in the principal keyword patterns across the two databases.

### 3.8 Conceptual structure and thematic evolution

Multiple correspondence analysis identified five broad conceptual clusters (**Figure 8A**). One cluster comprised osteogenesis, immunomodulation, angiogenesis, antibacterial, bone repair, and osteoimmunology. A second cluster included tissue engineering, wound healing, drug delivery, nanomaterial, exosome, macrophage, and macrophage polarization. A third cluster contained periodontal regeneration, periodontitis, hydrogel, anti-inflammatory, reactive oxygen species, autophagy, electrospinning, and 3D printing. The remaining two clusters were characterized by biomaterial, extracellular vesicle, stem cells, regenerative medicine, and nanomedicine, and by osseointegration, titanium, surface modification, and polyetheretherketone, respectively.

**Figure 8:**
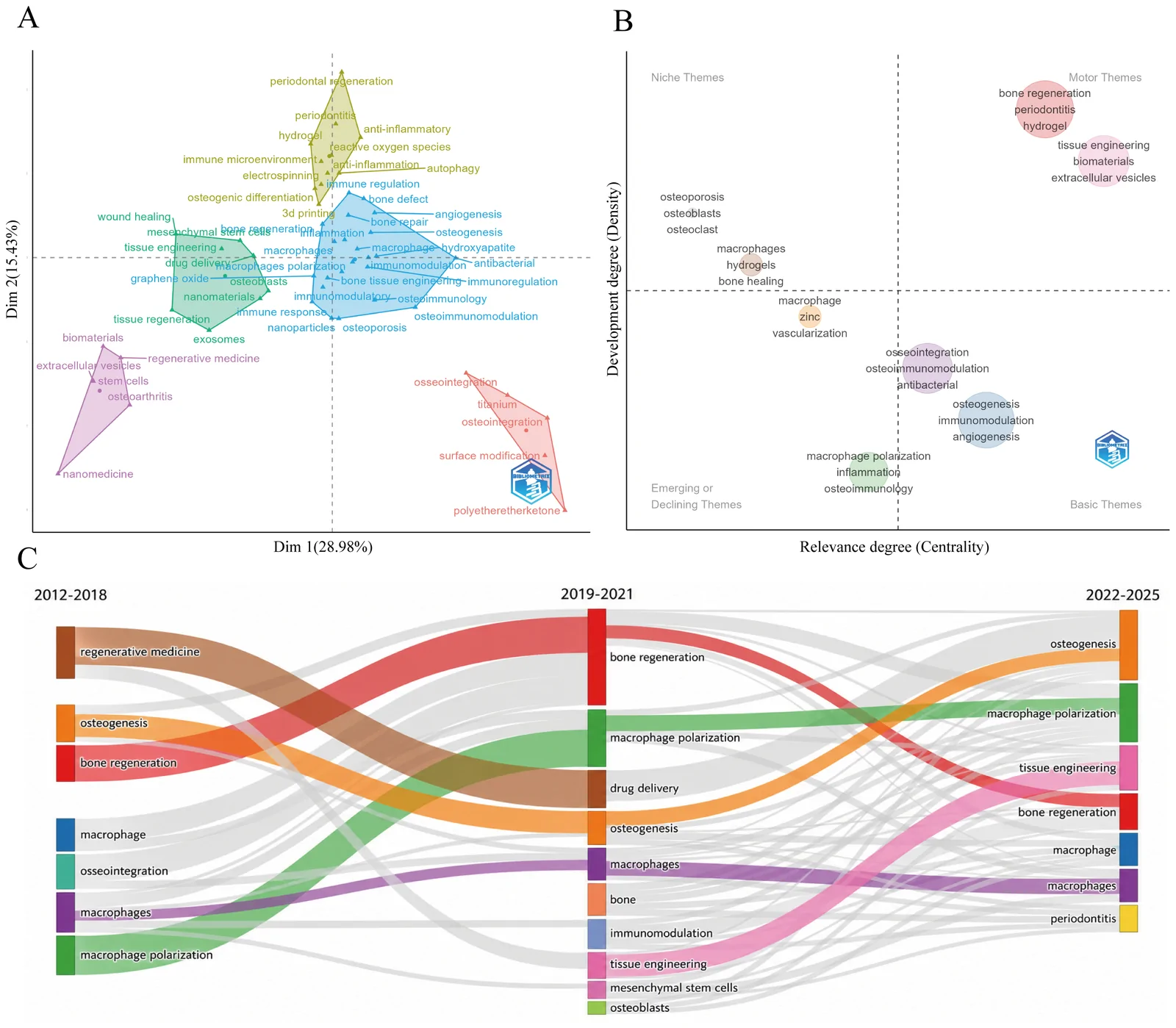
Conceptual structure, thematic development, and temporal evolution of research topics. **(A)** Conceptual structure map generated using multiple correspondence analysis of author keywords. The proximity of terms represents conceptual similarity, and colored polygons indicate keyword clusters. **(B)** Thematic map based on cluster centrality and density. Motor themes are located in the upper-right quadrant, niche themes in the upper-left quadrant, emerging or declining themes in the lower-left quadrant, and basic themes in the lower-right quadrant. Bubble size represents the frequency of the corresponding thematic cluster. **(C)** Thematic evolution across three periods: 2012–2018, 2019–2021, and 2022–2025. The size of each node represents theme frequency, while link width indicates the degree of continuity or shared keywords between themes in consecutive periods.

The thematic map further differentiated keyword clusters according to centrality and density (**Figure 8B**). The bone regeneration–periodontitis–hydrogel and tissue engineering–biomaterial–extracellular vesicle clusters were located in the motor-theme quadrant. The osteogenesis–immunomodulation–angiogenesis and osseointegration–osteoimmunomodulation–antibacterial clusters were positioned in the basic-theme quadrant. The osteoporosis–osteoblasts–osteoclast and macrophage–hydrogel–bone healing clusters occupied the niche-theme quadrant, whereas macrophage–zinc–vascularization and macrophage polarization–inflammation–osteoimmunology were located in the emerging-or-declining quadrant. Because the lower-left quadrant does not distinguish emerging from declining themes on its own, these clusters were considered together with their temporal trajectories in **Figure 8C**.

The thematic evolution map showed changes in theme composition across the three study periods (**Figure 8C**). During 2012–2018, major themes included regenerative medicine, osteogenesis, bone regeneration, macrophage-related research, osseointegration, and macrophage polarization. During 2019–2021, additional themes included drug delivery, immunomodulation, tissue engineering, mesenchymal stem cells, and osteoblasts, while bone regeneration, osteogenesis, and macrophage polarization remained represented. During 2022–2025, prominent themes included osteogenesis, macrophage polarization, tissue engineering, bone regeneration, and periodontitis. Bone regeneration and osteogenesis were present across all three periods, while macrophage polarization remained represented through the most recent period. Overall, tissue engineering and periodontitis were comparatively more prominent in 2022–2025, alongside the continued representation of bone regeneration, osteogenesis, and macrophage polarization.

### 3.9 Reference co-citation structure and citation-burst dynamics

Among the 25 references with the strongest citation bursts, most burst periods began after 2017 (**Figure 9A**). Chen et al. (2016), published in Materials Today, showed the strongest citation burst (strength = 23.93), lasting from 2018 to 2021. This was followed by Hotchkiss et al. (2016) in Acta Biomaterialia (strength = 13.44; 2017–2021), Chen et al. (2017) in ACS Nano (strength = 11.98; 2018–2022), and Pajarinen et al. (2019) in Biomaterials (strength = 11.95; 2020–2025). Earlier burst references included Ma et al. (2014), whose burst occurred primarily during 2016–2019. Several references remained in active burst periods through 2025, including Pajarinen et al. (2019), Lee et al. (2019), Zhu et al. (2021), and Dai et al. (2021).

**Figure 9:**
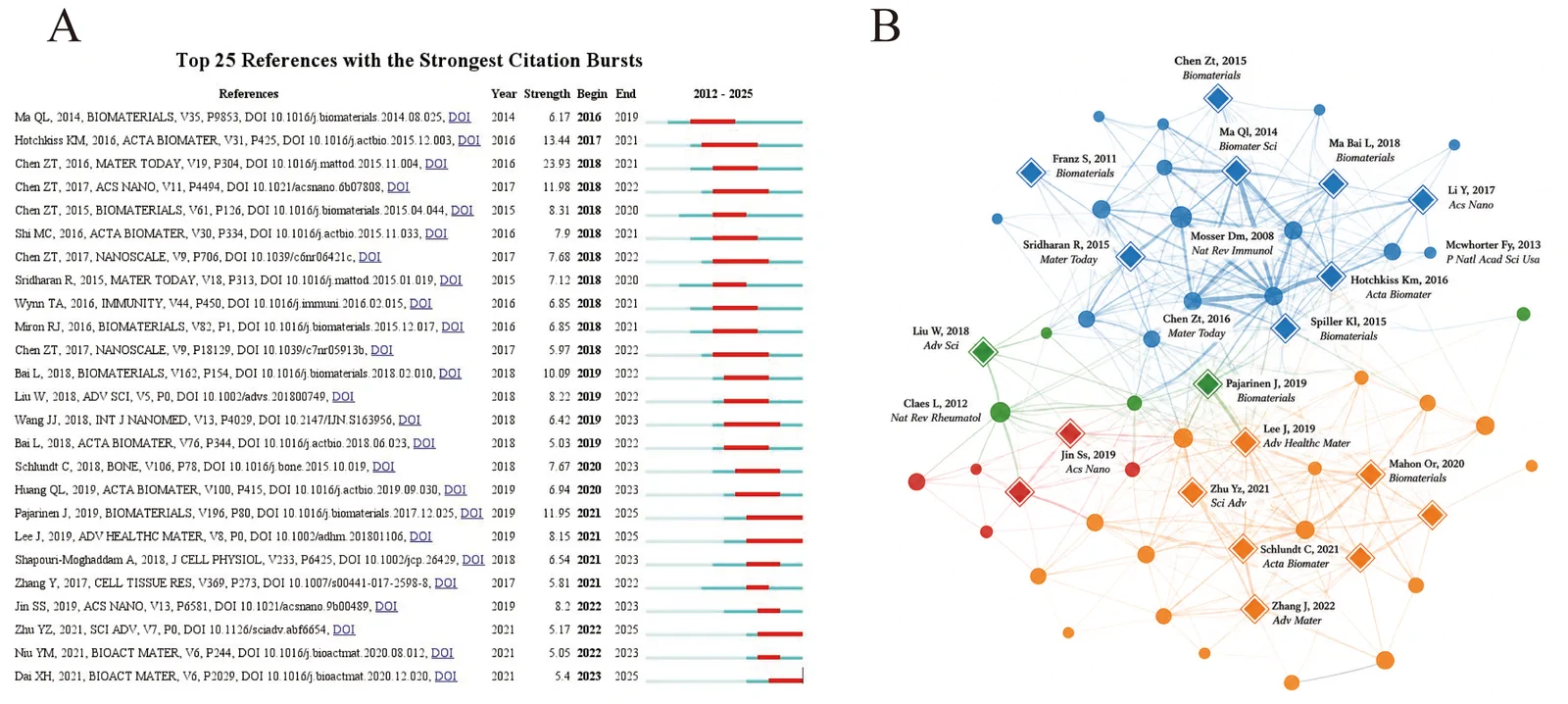
Citation-burst dynamics and reference co-citation structure of the field. **(A)** Top 25 references with the strongest citation bursts during 2012–2025. The red segments indicate the periods in which each reference experienced a marked increase in citation activity, while burst strength, beginning year, and ending year are shown alongside each reference. **(B)** Reference co-citation network of the core cited literature. Each node represents a cited reference, node size is proportional to its local citation frequency within the analyzed dataset, and link width reflects co-citation strength. Node colors indicate the four co-citation clusters identified using the Louvain community-detection algorithm, while diamond-shaped nodes denote references included in the citation-burst analysis. The network includes 63 core references and displays the 250 strongest co-citation links.

The reference co-citation network comprised four major clusters (**Figure 9B**). The largest cluster included Mosser et al. (2008), Franz et al. (2011), Spiller et al. (2015), Chen et al. (2016), and Hotchkiss et al. (2016). Chen et al. (2016) formed the most prominent node in the network and had the highest local citation frequency. A second cluster included Pajarinen et al. (2019), Lee et al. (2019), Zhu et al. (2021), Schlundt et al. (2021), and Zhang et al. (2022), while two smaller clusters comprised additional references represented in the co-citation network. Citation-burst references, indicated by diamond-shaped nodes, were distributed across all four clusters, with a greater concentration in the two larger clusters.

## 4 Discussion

The present study reveals a conceptual transition in nanomaterial-enabled bone regeneration, shifting from primarily osteoconductive and structural-support strategies toward active regulation of the osteoimmune microenvironment. This transition is reflected by the rapid expansion of the field, with 77.2% of the analyzed literature published during 2022–2025, and by the increasing integration of immune regulation with angiogenesis, tissue engineering, osseointegration, and disease-specific regenerative approaches. Although bone regeneration and osteogenesis remain foundational themes, the prominent positions of macrophage, macrophage polarization, immunomodulation, and osteoimmunomodulation indicate that immune regulation has become a central design consideration in contemporary regenerative materials research. The emergence of tissue engineering and periodontitis as more prominent themes during the most recent period further suggests that the field is extending beyond generalized bone repair toward complex biological environments characterized by inflammatory, microbial, and tissue-specific constraints. Together, these findings indicate that nanomaterial-enabled bone regeneration is evolving toward multifunctional and context-dependent platforms that coordinate material properties with immune, vascular, and skeletal responses.

This conceptual transition is also reflected in the intellectual structure identified by the co-citation and citation-burst analyses. Importantly, the foundational studies represented in this structure did not all focus specifically on functional nanomaterials; rather, they established material–immune principles that subsequently informed nanoengineered regenerative strategies. Chen et al. established osteoimmunomodulation as a design principle for bone-regenerative materials, emphasizing the capacity of implanted materials to regulate the local immune environment (21). Hotchkiss et al. subsequently demonstrated that titanium surface topography and wettability alter macrophage activation and cytokine production, establishing a direct link between interface physicochemistry and host immune responses (22). Chen et al. extended this concept to nanoengineered surfaces and showed that variations in nanotopography and surface chemistry modify inflammatory, osteogenic, angiogenic, and osteoclastic signaling (23). Pajarinen et al. further positioned macrophage-mesenchymal stromal-cell communication within the cellular framework of bone healing (24). Collectively, the strong representation of these references in our citation-burst and co-citation analyses traces a progression from viewing regenerative materials primarily in terms of structural and osteogenic performance toward considering them as active regulators of immune–skeletal interactions. This intellectual progression provides a mechanistic context for the current prominence of macrophage regulation and osteoimmunomodulation in the contemporary keyword structure.

The high frequency of macrophage polarization in the present analysis reflects the increasing mechanistic resolution of macrophage biology. The bibliometric term encompasses multiple changes in differentiation, metabolism, transcriptional state, phagocytic function, and communication with skeletal cells, and therefore represents a broader spectrum of functional states than a simple binary polarization framework would imply. TGFβ priming can reprogram TNF responses in macrophages and drive a non-canonical osteoclastogenic program, demonstrating direct plasticity between inflammatory signaling and bone-resorptive cell fate (25). Macrophage-derived signals can also act systemically; osteopontin released from adipose-tissue macrophages has been shown to influence bone-marrow macrophage metabolism and osteoclast activity (26). Metabolic intermediates provide another regulatory layer, with succinate coordinating macrophage inflammatory responses, endothelial-cell activity, stromal-cell migration, and osteogenic differentiation during bone healing (27). Osteal macrophage function also includes clearance-dependent regulation, as osteoblast apoptosis can stimulate macrophage efferocytosis, vascular responses, osteoprogenitor expansion, and bone formation (28). At a more fundamental level, macrophages rapidly reorganize transcription through RNA-polymerase-II pause release during successive efferocytosis, illustrating the speed with which functional macrophage states can be reprogrammed following environmental cues (29). These observations give biological depth to the prominence of macrophage-related keywords in our dataset and suggest that their bibliometric visibility reflects the growing mechanistic diversity of macrophage-related research in bone regeneration. Future nano-osteoimmunology studies will benefit from characterizing macrophage responses through temporal state transitions, metabolic programs, efferocytic capacity, osteoclastogenic potential, and intercellular signaling within the broader multicellular osteoimmune environment.

The prominence of hydrogels and tissue engineering in the thematic structure further indicates that nanomaterial-enabled regeneration is increasingly being developed within composite and dynamically interacting material–host systems. Because nanomaterial components are commonly incorporated into hydrogel, scaffold, membrane, or implant systems, the properties of the surrounding matrix provide an important mechanistic context for interpreting nanomaterial-enabled regenerative effects. Hydrogel crosslinking can alter stiffness, degradation, cell infiltration, macrophage states, fibroblast activity, and macrophage–fibroblast communication, demonstrating that physical material properties directly reorganize host-cell interactions (30). In bone regeneration, tuning the composition of macroporous scaffolds has been shown to modify macrophage subpopulations and macrophage communication with stromal and endothelial cells through signaling networks associated with inflammation resolution, angiogenesis, and osteogenesis (31). Self-adaptive scaffolds can similarly coordinate macrophage regulation with endogenous stem-cell recruitment and vascularized osteogenesis in large cranial defects (32). Host immune history also modifies regenerative outcomes: pre-existing sensitization to polyethylene glycol changes immune-cell recruitment and the amount and morphology of regenerated bone following PEG-hydrogel implantation, with additional sex-dependent effects (33). Temporal mechanics provide another level of control, as hydrogels with programmed spatiotemporal mechanical cues can regulate stem-cell-assisted bone regeneration across successive phases of healing (34). These findings support a material–host interaction model in which composition, architecture, mechanical properties, degradation, host immune history, and temporal presentation of biological cues collectively determine the regenerative response. Within nanomaterial-enabled systems, these broader material properties shape the context in which nanoscale cues act and therefore help explain the close association of hydrogels, tissue engineering, immunomodulation, and bone regeneration observed in our thematic analyses.

The relationships among tissue engineering, angiogenesis, osseointegration, and osteogenesis also indicate that nanomaterial-enabled regeneration operates within a broader multiscale architectural and mechanical environment. Patient-matched scaffold-guided regeneration has demonstrated that numerical optimization of scaffold architecture, mechanical performance, surface treatment, and osteoinductive components can support reconstruction of load-bearing segmental defects in a large-animal model (35). Metamaterial scaffolds with stage-dependent mechanical behavior can generate tissue strains that enhance calcium-channel activity, HIF-1α signaling, osteogenesis, and angiogenesis during critical-defect repair (36). Remotely activated magnetoelectric membranes provide a complementary strategy in which nanoscale core–shell structures convert magnetic stimulation into electrical cues that promote bone regeneration even under inflammatory or osteogenesis-suppressed conditions (37). Together, these studies illustrate that nanoscale functional cues are embedded within larger structural and mechanical systems that determine tissue infiltration, force transmission, vascularization, and implant integration. Nanoscale chemistry, topography, catalytic activity, and payload delivery therefore need to be considered in conjunction with the larger architectural and mechanical context in which these regenerative processes occur. The connectivity of these themes in our bibliometric network is consistent with an increasing convergence between nanoscale functional design and tissue-level regenerative engineering.

The increasing prominence of periodontitis during 2022–2025 represents a particularly relevant development because periodontal bone loss combines microbial stimulation, chronic inflammation, immune-cell activation, osteoclastogenesis, and regenerative failure within a spatially constrained environment. Lithiated porous silicon nanowires have demonstrated controlled ion release and enhanced regeneration of complex periodontal tissues, providing a direct example of nanoengineered physicochemical regulation in periodontal repair (38). Nanoparticulate scavenging of cell-free DNA can suppress inflammatory signaling, regulate mononuclear phagocytes, and reduce alveolar bone loss, linking nanoscale molecular sequestration with immune regulation (39). Metal–phenolic nanozyme platforms have further integrated antibacterial activity, reactive-oxygen-species scavenging, and osteogenic regulation in experimental periodontitis (40). Sulfated-chitosan-modified CuS nanoclusters provide another multifunctional strategy combining bacterial control with alveolar-bone regeneration (41). At the cellular level, fibroblast-derived complement C3 can promote inflammatory macrophage polarization and osteoclast differentiation, revealing direct stromal–immune–skeletal coupling within periodontal inflammation (42). These mechanistic and material studies provide a biological explanation for the recent emergence of periodontitis in our thematic analysis. More broadly, this trend suggests that nanomaterial-enabled bone regeneration is increasingly being evaluated in disease-specific microenvironments, where successful repair requires simultaneous control of microbial burden, inflammation, oxidative stress, and bone remodeling rather than enhancement of osteogenesis alone. Periodontal regeneration therefore provides a representative model for the shift from generalized regenerative strategies toward context-dependent, multifunctional osteoimmune regulation.

Comparable complexity is present in infected and metabolically compromised skeletal environments. In Staphylococcus aureus osteomyelitis, macrophage-derived amphiregulin drives myofibroblast differentiation of marrow adipogenic-lineage precursors around abscesses, restricting local vascular perfusion and antimicrobial access (43). Biodegradable Janus sonozymes have addressed infected critical-sized defects through stage-dependent regulation of reactive oxygen species, coupling antimicrobial activity with subsequent regenerative functions (44). Magneto-piezoelectric nanoparticle-enhanced scaffolds have further identified Icam1-positive macrophages and impaired oxidative phosphorylation as a targetable immune-metabolic component of infectious bone-defect regeneration (45). In diabetic defects, glucose- and ROS-responsive hydrogel–nanozyme systems can simultaneously modulate local glucose burden, oxidative stress, macrophage activity, angiogenesis, osteogenesis, and osteoclast activity (46). Nanomaterial-enabled immune regulation can also operate outside the local defect: colon-targeted postbiotic nanoparticles have been shown to suppress intestinal macrophage inflammation and influence systemic bone remodeling through the gut–bone axis (47). These studies extend the osteoimmune framework across local infection, metabolic dysfunction, and systemic inflammatory signaling. Importantly, they also indicate that immunomodulation is not a uniform therapeutic objective across regenerative settings; the required biological response depends on the specific combination of microbial burden, redox imbalance, metabolic disturbance, vascular dysfunction, and abnormal bone remodeling. Disease-specific microenvironments should therefore be considered not simply as background conditions, but as determinants of how nanomaterial-enabled regenerative systems are designed and evaluated.

Although macrophages occupy a central position in our keyword structure, the osteoimmune network extends beyond a single cell type. Eosinophils contribute to bone repair through cytokine secretion and osteoblast regulation, whereas mast cells influence angiogenesis, extracellular matrix remodeling, and osteogenic signaling (48, 49). T cells provide adaptive immune regulation by modulating inflammatory resolution and osteoclastogenesis, and their interactions with macrophages and mesenchymal stromal cells further complicate immune regulation during skeletal repair (50). The relatively lower bibliometric visibility of these immune populations may reflect differences in research maturity, terminology usage, and incorporation into nanomaterial studies rather than their biological relevance alone. Future investigations should therefore integrate multicellular immune networks, including innate and adaptive immune components, into the design and evaluation of osteoimmunomodulatory materials. Such expansion will be particularly important for understanding how nanomaterial-enabled systems coordinate interactions among immune, stromal, vascular, and skeletal compartments during different stages of regeneration.

Future nanomaterial platforms will likely evolve from static multifunctional systems toward programmable regenerative systems that respond dynamically to changing biological conditions. The next generation of osteoimmunomodulatory materials may incorporate spatiotemporal control, sequential therapeutic delivery, and adaptive responses to inflammatory and regenerative signals rather than simply combining multiple functions within a single construct. Biomaterial systems capable of modulating macrophage metabolism, controlling reactive oxygen species, regulating vascular responses, and coordinating osteogenesis represent early examples of this transition. However, increasing functional complexity also introduces challenges regarding material stability, reproducibility, degradation behavior, and biological predictability. Therefore, future advances will require not only improved material functionality but also a clearer understanding of how specific material cues translate into defined cellular and tissue-level outcomes. Such mechanistic integration will be essential for developing nanomaterial-enabled regenerative platforms that are both biologically effective and clinically feasible.

Despite substantial advances in nanomaterial-enabled osteoimmunomodulation, several translational barriers remain before these strategies can be broadly implemented clinically. First, long-term biosafety and degradation behavior require careful evaluation because nanoscale materials may exhibit complex interactions with immune cells, metabolites, and clearance pathways beyond the initial regenerative phase. Second, the therapeutic effects observed in small-animal models may not fully capture the complexity of human bone repair, where differences in immune status, aging, metabolic conditions, defect size, and mechanical loading can substantially influence outcomes. Third, manufacturing scalability, batch-to-batch consistency, sterilization compatibility, and regulatory requirements remain critical considerations for clinical translation of multifunctional nanomaterial systems. Moreover, increasing material complexity may create challenges in defining mechanism-based quality criteria and predicting patient-specific responses. Future research should therefore integrate advanced material design with standardized evaluation frameworks, clinically relevant models, and longitudinal safety assessment. A successful transition from experimental nanomaterial-enabled regeneration toward clinical application will require balancing multifunctionality with simplicity, controllability, and reproducibility.

## 5 Limitations

Several limitations should be acknowledged. First, the analysis included only English-language articles and reviews indexed in the Web of Science Core Collection and Scopus; therefore, relevant studies indexed in other databases or published in other languages may have been missed. Because the search strategy was based on predefined terminology, studies using emerging or less standardized expressions may have been less readily identified. Second, despite systematic data cleaning and harmonization, residual inconsistencies in author names, institutional affiliations, and keyword terminology may have affected productivity rankings and network structures. Third, bibliometric analyses based on keyword frequency and co-occurrence may preferentially capture highly studied concepts and research communities. Keyword-based analyses primarily reflect patterns of terminology use and bibliometric visibility and may not fully represent the multidimensional biological complexity of the underlying scientific topics. Therefore, the prominence of macrophage-related terms in our analysis reflects current research emphasis and may not fully capture the diversity of osteoimmune regulation. Emerging or less frequently studied immune populations, including neutrophils and B cells, may receive less bibliometric visibility because of differences in research maturity, terminology, and incorporation into nanomaterial studies. Citation-based indicators were interpreted with consideration of their dependence on publication age and citation exposure, as these measures may favor earlier publications with longer citation windows. Fourth, reference co-citation and citation-burst analyses relied on cited-reference data from WoSCC, which may not fully represent the intellectual structure of the combined WoSCC–Scopus dataset. Finally, network clustering and keyword forecasting depend on analytical settings and historical occurrence patterns; these results should therefore be interpreted as descriptive structures and directional trends rather than causal relationships or precise predictions. In particular, keyword-based projections do not account for unexpected technological breakthroughs, changes in clinical priorities, or shifts in regulatory environments.

## 6 Conclusion

This study provides an integrated mapping of the scientific and intellectual landscape of nanomaterial-enabled bone regeneration from an osteoimmune perspective. The findings indicate that the field is evolving from conventional structural and osteogenic strategies toward multifunctional approaches that actively regulate immune–material interactions and the regenerative microenvironment. Macrophage-related research, immunomodulation, and osteoimmunomodulation are prominent within this transition, alongside increasing integration with angiogenesis, tissue engineering, osseointegration, antibacterial strategies, and disease-specific regenerative approaches. Future progress will depend on improving the mechanistic understanding of multicellular immune regulation, developing programmable and biologically predictable materials, and establishing clinically relevant validation frameworks to facilitate the translation of nanomaterial-enabled bone regeneration strategies.

## Data Availability

The bibliographic data analyzed in this study were obtained from the Web of Science Core Collection and Scopus. The complete database-specific search strategies and literature-screening information are provided in **Supplementary File 1**. Additional data supporting the findings of this study are available from the corresponding author upon reasonable request.
