# Supplementary material for: Functional nanomaterials for immunomodulation in bone regeneration: a bibliometric analysis of the osteoimmune research landscape
Source: Front Immunol. 2026 Oct 28;17:1978326. doi: 10.3389/fimmu.2026.1978326 (PMC13752172; doi:10.3389/fimmu.2026.1978326)
Supplement: Supplementary file 1 [file Supplementaryfile1.docx]

Supplementary Material

**Supplementary Material S1. Database search strategy**

The final database searches were conducted on 6 May 2026, with the publication period restricted to records published through 31 December 2025. Search concepts were organized into three blocks: immune or osteoimmune regulation, nanomaterials, and bone regeneration or osseointegration. Terms within each block were combined with OR, and the three blocks were linked with AND. No language or document-type restriction was applied to the initial retrieval.

**Table S1. Summary of database searches**

| **Database** | **Coverage** | **Search field** | **Export format** | **Initial records** |
| --- | --- | --- | --- | --- |
| Scopus | Inception to 31 Dec 2025 | TITLE-ABS-KEY | CSV | 849 |
| Web of Science Core Collection | Inception to 31 Dec 2025 | TS | Plain text; full record and cited references | 804 |

**Supplementary Material S1.1 Scopus search strategy**

**Search field: TITLE-ABS-KEY**

TITLE-ABS-KEY(
 (osteoimmun* OR immunomodulat* OR immunoregulat* OR "immune modulation" OR
 "immune regulation" OR "immune microenvironment*" OR
 "osteoimmune microenvironment*" OR "inflammatory microenvironment*" OR
 "macrophage polarization" OR "macrophage polarisation" OR
 "M1 macrophage*" OR "M2 macrophage*")
 AND
 (nanomaterial* OR nanoparticle* OR nanomedicine OR nanocarrier* OR
 nanocomposite* OR nanofiber* OR nanofibre* OR nanotube* OR nanosheet* OR
 nanostructur* OR nanoscaffold* OR nanocoating* OR nanogel* OR nanozyme* OR
 "nano-hydroxyapatite" OR nanohydroxyapatite)
 AND
 ("bone regeneration" OR "bone repair" OR "bone defect*" OR "bone healing" OR
 "fracture healing" OR osteogenesis OR osteogenic OR
 "bone tissue engineering" OR osseointegration OR "guided bone regeneration")
)

**Records retrieved before filters: 849.**

**Supplementary Material S1.2 Web of Science Core Collection search strategy**

**Search field: TS (Topic).**

TS=(
 (osteoimmun* OR immunomodulat* OR immunoregulat* OR "immune modulation" OR
 "immune regulation" OR "immune microenvironment*" OR
 "osteoimmune microenvironment*" OR "inflammatory microenvironment*" OR
 "macrophage polarization" OR "macrophage polarisation" OR
 "M1 macrophage*" OR "M2 macrophage*")
 AND
 (nanomaterial* OR nanoparticle* OR nanomedicine OR nanocarrier* OR
 nanocomposite* OR nanofiber* OR nanofibre* OR nanotube* OR nanosheet* OR
 nanostructur* OR nanoscaffold* OR nanocoating* OR nanogel* OR nanozyme* OR
 "nano-hydroxyapatite" OR nanohydroxyapatite)
 AND
 ("bone regeneration" OR "bone repair" OR "bone defect*" OR "bone healing" OR
 "fracture healing" OR osteogenesis OR osteogenic OR
 "bone tissue engineering" OR osseointegration OR "guided bone regeneration")
)

**Records retrieved before filters: 804.**

**Supplementary Material S2. Merged study-selection process**

All initially retrieved records were exported and merged. Duplicate records were identified primarily by DOI; records without a DOI were compared using title, author names, source title, and publication year. Suspected duplicates were manually reviewed. Eligibility exclusions were then counted in the deduplicated dataset, ensuring that overlapping records were counted only once.

**Table S2. Final study-selection counts**

| **Stage** | **Description** | **Excluded** | **Remaining** |
| --- | --- | --- | --- |
| Identification | Records retrieved from Scopus (n = 849) and WoSCC (n = 804) | — | 1,653 |
| Deduplication | Duplicate records removed after merging | 614 | 1,039 |
| Language screening | Non-English publications excluded | 14 | 1,025 |
| Document-type screening | Records other than original articles or reviews excluded | 19 | 1,006 |
| Final inclusion | Publications included in bibliometric and visualization analyses | — | 1,006 |

**Supplementary Material S3. Eligibility criteria**

Eligible records were English-language original research articles or reviews published through 31 December 2025. Duplicate records were removed before language and document-type screening. The final analytical set comprised records meeting these criteria after manual deduplication.

**Supplementary Material S4. Sensitivity analysis of WoSCC and Scopus**

To evaluate the robustness of the combined-database approach, author-keyword patterns were examined separately in WoSCC and Scopus using the same eligibility criteria and keyword-harmonization procedures as in the primary analysis. Before frequency ranking, synonymous terms and obvious lexical variants, including singular and plural forms, were consolidated under the same preferred terms. The 20 most frequent author keywords were then identified independently for each database, and concordance was assessed using top-20 keyword overlap, the Jaccard similarity coefficient, and Spearman’s rank correlation for shared keywords.

Nineteen of the 20 most frequent author keywords were shared between WoSCC and Scopus, corresponding to a top-20 overlap of 95% and a Jaccard similarity coefficient of 0.905. The rankings of the shared keywords were positively correlated (Spearman’s ρ = 0.802, P < 0.001), indicating substantial consistency in the principal author-keyword patterns between the two databases. These findings indicate that the major keyword patterns identified in the combined analysis were broadly robust to database source.

**Table S3. Comparison of the top 20 author keywords between WoSCC and Scopus**

| **Rank** | **WoSCC Top 20** | **Scopus Top 20** |
| --- | --- | --- |
| 1 | bone regeneration | bone regeneration |
| 2 | immunomodulation | immunomodulation |
| 3 | macrophage | osteogenesis |
| 4 | osteogenesis | macrophage |
| 5 | macrophage polarization | tissue engineering |
| 6 | tissue engineering | macrophage polarization |
| 7 | osteoimmunomodulation | hydrogel |
| 8 | nanoparticle | nanoparticle |
| 9 | osseointegration | biomaterial |
| 10 | biomaterial | drug delivery |
| 11 | antibacterial | inflammation |
| 12 | hydrogel | osteoimmunomodulation |
| 13 | mesenchymal stem cells | osseointegration |
| 14 | periodontitis | angiogenesis |
| 15 | inflammation | exosome |
| 16 | angiogenesis | mesenchymal stem cells |
| 17 | drug delivery | extracellular vesicle |
| 18 | nanomaterial | antibacterial |
| 19 | regenerative medicine | regenerative medicine |
| 20 | exosome | periodontitis |

**Note:** Nineteen keywords were shared between the two top-20 lists (overlap = 95%; Jaccard similarity coefficient = 0.905). Rank-order agreement among the shared keywords was assessed using Spearman’s rank correlation (ρ = 0.802, P < 0.001).
